# Supplementary figures and images for: 3,6’-dithiopomalidomide reduces neural loss, inflammation, behavioral deficits in brain injury and microglial activation
Source: eLife. 2020 Jun 26;9:e54726. doi: 10.7554/eLife.54726 (PMC7375814; doi:10.7554/eLife.54726)

Sham + DP (0.5 mg/kg) -5hr

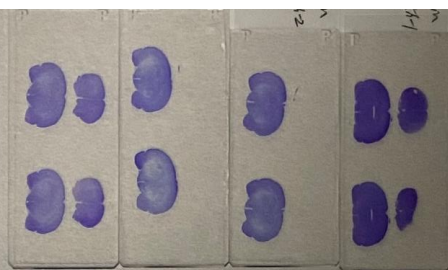

Sham

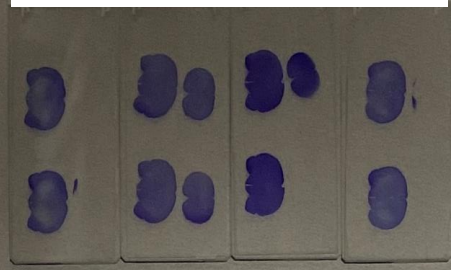

TBI + Veh

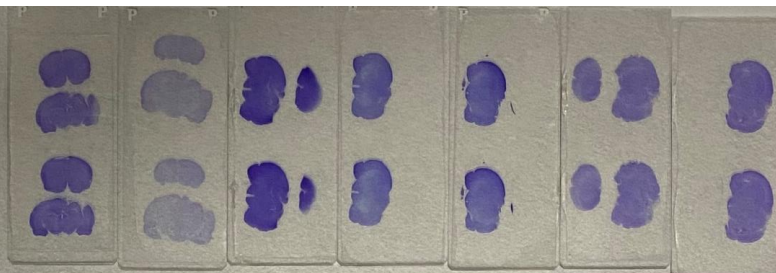

TBI + Pom (0.1 mg/kg) -5hr

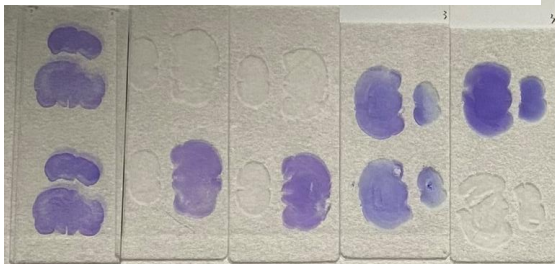

TBI + Pom (0.5 mg/kg) -5hr

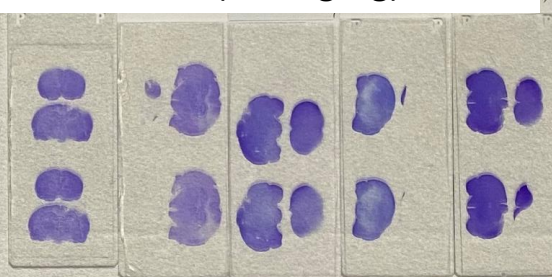

TBI + DP (0.5 mg/kg) -5hr

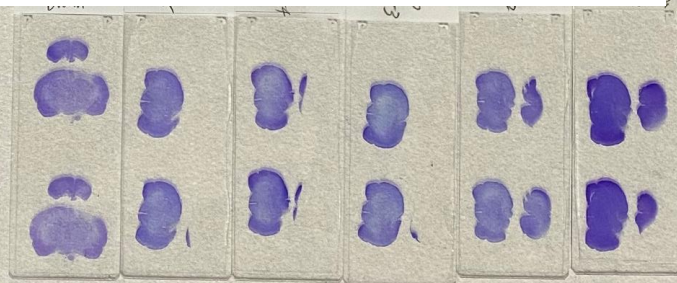

TBI + DP (0.5 mg/kg) -7hr

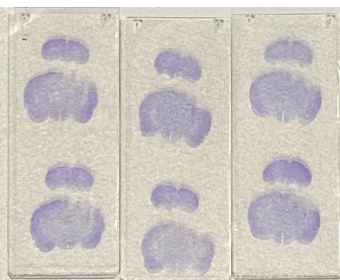

TBI + DP (0.1 mg/kg) -5hr

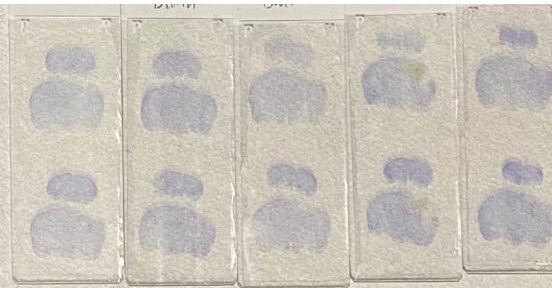

Supplement: Figure 2—source data 1. [file elife-54726-fig2-data1.pdf]

Sham + DP (0.5 mg/kg) -5hr

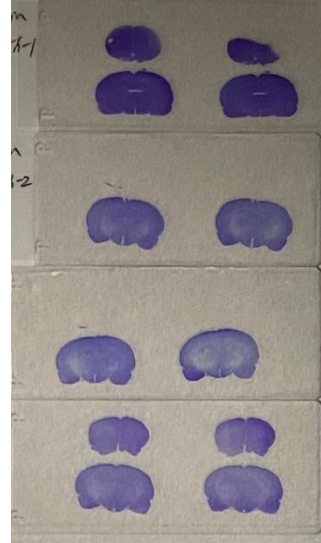

Sham

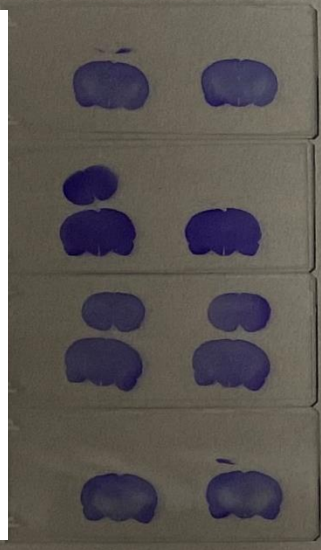

TBI + Veh

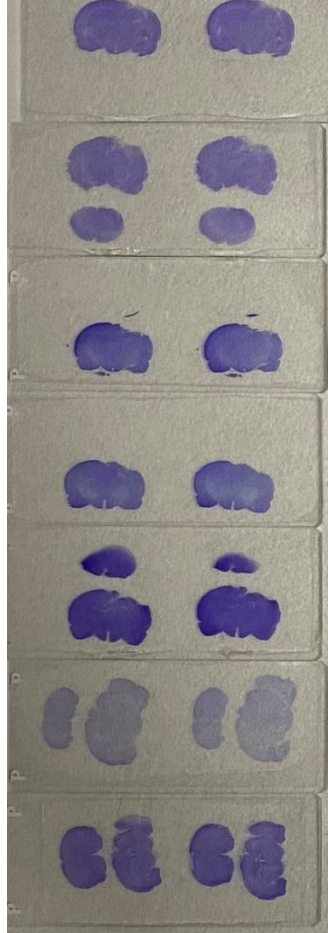

TBI + Pom (0.1 mg/kg) -5hr

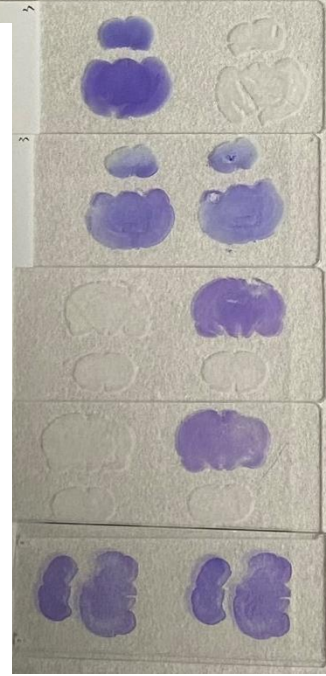

TBI + Pom (0.5 mg/kg) -5hr

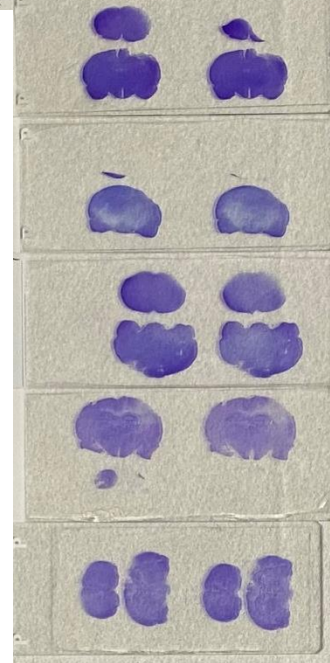

TBI + DP (0.5 mg/kg) -5hr

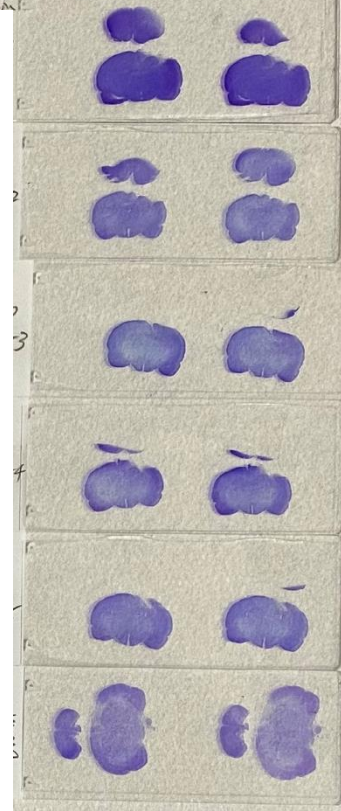

TBI + DP (0.5 mg/kg) -7hr

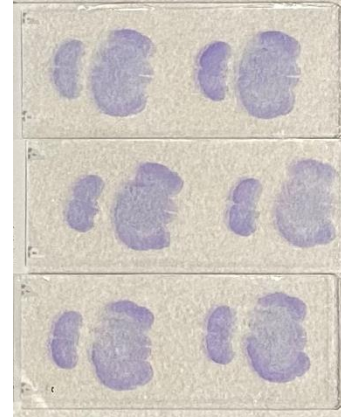

TBI + DP (0.1 mg/kg) -5hr

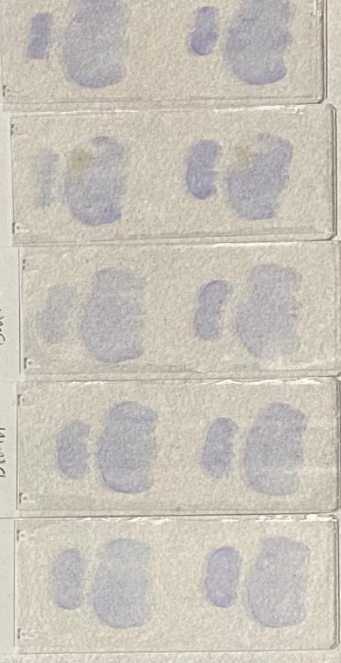

Supplement: Figure 3—source data 1. [file elife-54726-fig3-data1.pdf]
