# Supplementary material for: 3,6’-dithiopomalidomide reduces neural loss, inflammation, behavioral deficits in brain injury and microglial activation
Source: eLife. 2020 Jun 26;9:e54726. doi: 10.7554/eLife.54726 (PMC7375814; doi:10.7554/eLife.54726)
Supplement: Figure 13—source data 1. [file elife-54726-fig13-data1.docx]

**Raw data:** DP and Pom mitigate α-synuclein-induced losses of dopaminergic neurons and neurites as well as microglial cell activation in primary cultures (Fig. 13)

- **Immunostaining**
