# Supplementary material for: 3,6’-dithiopomalidomide reduces neural loss, inflammation, behavioral deficits in brain injury and microglial activation
Source: eLife. 2020 Jun 26;9:e54726. doi: 10.7554/eLife.54726 (PMC7375814; doi:10.7554/eLife.54726)
Supplement: Transparent reporting form [file elife-54726-transrepform.doc]

***eLife’s* transparent reporting form**

We encourage authors to provide detailed information *within their submission* to facilitate the interpretation and replication of experiments. Authors can upload supporting documentation to indicate the use of appropriate reporting guidelines for health-related research (see [EQUATOR Network](http://www.equator-network.org/ )), life science research (see the [BioSharing Information Resource](https://biosharing.org/)), or the [ARRIVE guidelines](http://www.plosbiology.org/article/info:doi/10.1371/journal.pbio.1000412) for reporting work involving animal research. Where applicable, authors should refer to any relevant reporting standards documents in this form.

If you have any questions, please consult our Journal Policies and/or contact us: [editorial@elifesciences.org](mailto:editorial@elifesciences.org).

**Sample-size estimation**

- You should state whether an appropriate sample size was computed when the study was being designed
- You should state the statistical method of sample size computation and any required assumptions
- If no explicit power analysis was used, you should describe how you decided what sample (replicate) size (number) to use

Please outline where this information can be found within the submission (e.g., sections or figure legends), or explain why this information doesn’t apply to your submission:

Sample size calculations – particularly for animal studies – as noted in the **Methods Section** (under ‘(iii) TBI studies’) were determined on the basis of “…….. both prior studies (Huang et al., 2018; Yang et al., 2016, 2015) and a power analysis (Charan and Kantharia 2013).” See also, under ‘In Vitro Studies: (i) DP and Pom activity in LPS stimulated RAW 264.7 cells’, the following: “….. sample size was selected on the basis of our prior studies (Tweedie et al., 2011; Tweedie et al., 2012) and a power analysis (Charan and Kantharia 2013)”.

Details of the power analysis for animals have been provided in our ‘Response to Reviewers’ document. Specifically: “We evaluated 5 rats for each group, based on (i) the statistical significance achieved in our prior TBI studies (see, for example: Huang et al. *Sci Rep* **8**: 2368) and, (ii), a power analysis based on a study conducted by Charan & Kantharia (2013) which suggests for any sample size, which keeps E (E = Total number of animals − Total number of groups) between 10 and 20 should be considered as adequate based on the ANOVA test. The E in our study is 24 (E=5*6-6) which is greater than 20, and means that adding further animals will not increase the chance of obtaining statistically significant results. Since our data showed clear statistical differences between Sham, TBI+Veh, Pom (0.5 and 0.1 mg/kg) and DP (0.1 and 0.5 mg/kg) groups, we did not add further animals to our behavioral experiments. As a general rule, we will add animals when there appears to be a clear trend that does not achieve statistical significance – or should an animal not be able to undertake a task (which would be noted in our manuscript, if occurring). In such a scenario we would include more animals according to 3R principle (reduction, replacement and refinement) for ethical use of animals.”

**Replicates**

- You should report how often each experiment was performed
- You should include a definition of biological versus technical replication
- The data obtained should be provided and sufficient information should be provided to indicate the number of independent biological and/or technical replicates
- If you encountered any outliers, you should describe how these were handled
- Criteria for exclusion/inclusion of data should be clearly stated
- High-throughput sequence data should be uploaded before submission, with a private link for reviewers provided (these are available from both GEO and ArrayExpress)

Please outline where this information can be found within the submission (e.g., sections or figure legends), or explain why this information doesn’t apply to your submission:

Our study was set up to evaluate, side-by-side (assessing equimolar concentrations/doses), two compounds ([i] a new drug candidate 3,6’-dithioPomalidomide (DP) and [ii] Pomalidomide (Pom) – an FDA approved drug used in the treatment of multiple myeloma) as a new treatment strategy for brain injury and neuroinflammation. Each piece of our studies was designed to be cross-validated and replicated by the next stage of the study.

For example, cellular studies were undertaken in RAW 264.7 cells challenged with the classical pro-inflammatory protein LPS to induce the generation and release of TNF in the presence and absence of DP and Pom. Studies in primary mixed culture of cells obtained from brain demonstrated that DP and Pom mitigated neuroinflammation induced by two classical disease-relevant proinflammatory proteins (alpha-synuclein and amyloid-beta) – thereby cross-validating the initial RAW cell studies by three separate measures. Additionally, First-in-animal studies evaluating the ability to mitigate the systemic and brain pro-inflammation induced by systemic administration of LPS to rodents fully cross-validate the initial RAW cell studies (with DP and Pom providing TNF-lowering actions). This study was then cross-validated in the clinically relevant TBI CCI rodent model, where DP in particular, but also a 5-fold higher dose of Pom, mitigated multiple injury-induced deficits (evaluating multiple factors associated with neuroinflammation, including TNF levels at both the protein and mRNA level, as well as behavioral end points).

Finally, this new Pom data is fully replicated in a separate study series of animals, in a small prior study (that is noted as the reference: Wang et al., 2016). The current study not only replicates our former small initial study of Pom, but substantially expands this – and more, importantly provides first-in-animal studies of a new more potent Pom analog (specifically, DP) designed and developed to mitigate neuroinflammation.

This continuous replication and cross-validation of data within and across cellular to more complicated animal studies is a cornerstone of the drug development process, and is noted in multiple places within our manuscript, and particularly in the Discussion.

**Statistical reporting**

- Statistical analysis methods should be described and justified
- Raw data should be presented in figures whenever informative to do so (typically when N per group is less than 10)
- For each experiment, you should identify the statistical tests used, exact values of N, definitions of center, methods of multiple test correction, and dispersion and precision measures (e.g., mean, median, SD, SEM, confidence intervals; and, for the major substantive results, a measure of effect size (e.g., Pearson's r, Cohen's d)
- Report exact p-values wherever possible alongside the summary statistics and 95% confidence intervals. These should be reported for all key questions and not only when the p-value is less than 0.05.

Please outline where this information can be found within the submission (e.g., sections or figure legends), or explain why this information doesn’t apply to your submission:

Statistical analyses are noted in the Methods Section, and specific details are provided in each legend associated with the various Figures.

(For large datasets, or papers with a very large number of statistical tests, you may upload a single table file with tests, Ns, etc., with reference to sections in the manuscript.)

**Group allocation**

- Indicate how samples were allocated into experimental groups (in the case of clinical studies, please specify allocation to treatment method); if randomization was used, please also state if restricted randomization was applied
- Indicate if masking was used during group allocation, data collection and/or data analysis

Please outline where this information can be found within the submission (e.g., sections or figure legends), or explain why this information doesn’t apply to your submission:

As noted within the Methods Section (under ‘TBI studies’), “…Following sham or TBI procedures, animals were randomly assigned to treatment or veh (control) groups”.

**Additional data files (“source data”)**

- We encourage you to upload relevant additional data files, such as numerical data that are represented as a graph in a figure, or as a summary table
- Where provided, these should be in the most useful format, and they can be uploaded as “Source data” files linked to a main figure or table
- Include model definition files including the full list of parameters used
- Include code used for data analysis (e.g., R, MatLab)
- Avoid stating that data files are “available upon request”

Please indicate the figures or tables for which source data files have been provided:

Figures 1 thru 13
